# Visualizing Antigen Specific CD4+ T Cells using MHC Class II Tetramers

**DOI:** 10.3791/1167

**Published:** 2009-03-06

**Authors:** Eddie A. James, Rebecca LaFond, Ivana Durinovic-Bello, William Kwok

**Affiliations:** Tetramer Core Laboratory, Benaroya Research Institute; Kwok Laboratory, Benaroya Research Institute; Nepom Laboratory, Benaroya Research Institute

## Abstract

Major histocompatibility complex (MHC) class II tetramers allow the direct visualization of antigen specific CD4+ T cells by flow cytometry.  This method relies on the highly specific interaction between peptide loaded MHC and the corresponding T-cell receptor.  While the affinity of a single MHC/peptide molecule is low, cross-linking MHC/peptide complexes with streptavidin increases the avidity of the interaction, enabling their use as staining reagents.  Because of the relatively low frequencies of CD4+ T cells (~1 in 300,000 for a single specificity) this assay utilizes an in vitro amplification step to increase its threshold of detection.  Mononuclear cells are purified from peripheral blood by Ficoll underlay.  CD4+ cells are then separated by negative selection using biotinylated antibody cocktail and anti-biotin labeled magnetic beads.  Using adherent cells from the CD4- cell fraction as antigen presenting cells, CD4+ T cells are expanded in media by adding an antigenic peptide and IL-2.  The expanded cells are stained with the corresponding class II tetramer by incubating at 37 C for one hour and subsequently stained using surface antibodies such as anti-CD4, anti-CD3, and anti-CD25.  After labeling, the cells can be directly analyzed by flow cytometry.  The tetramer positive cells typically form a distinct population among the expanded CD4+ cells.  Tetramer positive cells are usually CD25+ and often CD4 high.  Because the level of background tetramer staining can vary, positive staining results should always be compared to the staining of the same cells with an irrelevant tetramer.  Multiple variations of this basic assay are possible.  Tetramer positive cells may be sorted for further phenotypic analysis, inclusion in ELISPOT or proliferation assays, or other secondary assays.  Several groups have also demonstrated co-staining using tetramers and either anti-cytokine or anti-FoxP3 antibodies.

**Figure:**
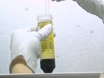


## Protocol

### 1. Peripheral blood mononuclear cell (PBMC) isolation

Obtain a blood sample – blood should be collected in syringes or blood tubes and anti-coagulated with heparin (1:50 ratio) to prevent clotting.  Expect a yield of about 1×10^6^ PBMC per mL of blood – about 40% of which will be CD4 positive (CD4+) T cells.
Aliquot the blood into 50 mL conical tubes, 25 mL per tube.  If blood has separated, gently mix before aliquoting to distribute the plasma
Add PBS, bringing the total volume to 40 mL and underlay by drawing up 11 mL of Ficoll, inserting into blood tube, and carefully removing the pipet Aid from the pipette.  The ficoll will slowly drain into the bottom of the tube.  Once the level has equalized, slowly remove the pipette from the blood tube and discard.
Cap the blood tubes and move into the Aerosolve canisters.  Firmly close the canisters and centrifuge at 900×g for 20 minutes – no brake.  It is critical that no brake is applied at the end of this spin as the braking force will disturb the layer of cells.
After the spin, the PBMCs should form a distinct white layer between the yellow plasma (above) and transparent ficoll (below).  Gently skim off white blood cell layer using a transfer pipette and transfer to new tube.  Some plasma and Ficoll may be drawn up with the PBMCs, but avoid drawing up any of the red cell layer.  Typically two blood tubes can be combined into one cell tube at this step to increase the pellet size.
Add PBS, bringing the total volume to 50 mL and centrifuge at 500×g for 15 minutes – low brake in aerosolve canisters.
Aspirate liquid using a Pasteur pipette, taking care not to disturb the pellet
Treat with hemolytic buffer by adding 5-6 mL to each tube and gently mixing to remove clumps.  Incubate for no more than 5 minutes.
Add PBS, bringing the total volume to 50 mL and centrifuge at 230×g for 10 minutes – low brake.  Aerosolve canisters are no longer needed for these slower spins.
Wash two times: Aspirate liquid using a Pasteur pipette, fill tube with PBS and centrifuge at 230×g for 10 minutes.
Prior to the final wash step, remove an aliquot of re-suspended cells, dilute with trypan blue, and count using a hemocytometer.  This cell count will dictate the reagent volumes used in the T cell separation step.

### 2. CD4+ T cell separation^*^

Aspirate liquid and add running buffer to bring the total volume up to 40 uL per 10 million cells.  Usually this requires 30 uL of buffer plus the residual pellet volume.
Transfer this volume to a 15 mL conical tube.
Add antibody cocktail from the CD4 isolation kit – 10 uL per 10 million cells, cap tube, and place on ice for 10 minutes
Remove tube from ice, remove cap, and add 30 uL of running buffer per 10 million cells
Add magnetic beads from the CD4 isolation kit – 20 uL per 10 million cells, cap tube, and place on ice for 15 minutes
Add ten volumes of running buffer to wash and centrifuge at 230×g for 10 minutes.
During the spin, set up the magnet and 5 mL polypropylene tube in your workspace
Aspirate liquid from cell pellet, add 2 mL running buffer and remove clumps using a transfer pipette
Using the same transfer pipette, transfer cells into the 5 mL tube and incubate in the magnet for 15 minutes
Carefully decant the CD4+ cell fraction into a marked 15 mL tube by inverting the magnet and emptying all but the last drop
Add another 2 mL of running buffer to the 5 mL tube and remove from magnet
Gently rinse the CD4- cells off the sides of tube and transfer to a marked 15 mL tube
Add 2 mL T cell medium to each tube, remove aliquots for cell counting and centrifuge at 230×g for 10 minutes.
Dilute cell aliquots with trypan blue and count using a hemocytometer.  These cell counts will dictate the number of wells used for the expansion culture step.

* Alternatively, MACS columns and beads (Miltenyi Biotec), the AutoMACS cell separator (Miltenyi Biotec), or Robosep cell separator (Stem Cell Technologies) can be used according to manufactures instructions in place of steps 2.2 – 2.11.

### 3. In vitro Expansion culture

Aspirate liquid from the CD4+ and CD4- cell pellets and, based on the cell counts, add culture media (typically 3 million CD4+ cells per mL and 10 million CD4- cells per mL works well for setting up the culture).  The expansion culture requires 3-5 million CD4- cells per well and 2-3 million CD4+ cells per well in a total volume of 1 mL culture media in a 48 well culture plate.  Typically, CD4+ cells are the limiting population
If desired, irradiate the CD4- cells (5000 Rads).  Aliquot CD4- cells into the appropriate number of wells on a 48 well plate by adding 300-500 μL to each.  Place the plate in a 37°C incubator for 1 hour.  The adherent CD4- cells will serve as antigen presenting cells in the expansion culture.
Remove the plate from the incubator.
Wash the wells by adding 500 uL of fresh media and gently pipetting up and down 12-20 times using a transfer pipet and removing all of the liquid.  This wash will leave behind a layer of adherent antigen presenting cells.
When the washing is complete, add just enough media to wet the bottom of each well (roughly 100 uL) – otherwise the adherent cells will dry out
Add 2-3 million CD4+ cells to each well.  If necessary, add additional media to bring the total volume in each well up to 1 mL.
Add the desired peptide to each well.  The typical concentration for stimulation is 10 ug/mL final.
Place plate in a 37°C incubator for 7 days.
After 7 days, add 50 uL of Hemogen IL-2 (or equivalent, such as recombinant IL-2, 10 IU/mL) to each well.
On each subsequent day, monitor cell growth using a microscope.  Feed wells that are not yet confluent by removing half of the media, adding fresh media and 50 uL of IL-2.  Split confluent wells by resuspending the cells, moving half of the cells to an empty well, adding fresh media and 50 uL of IL-2.  Return cells to incubator.
The expanding cells will be ready for tetramer after 13-15 days of culture.

### 4. Visualizing T cells by tetramer staining

Purchase or assemble tetramers to match the peptide/MHC combinations that match the stimulated CD4+ T cells (see discussion for sources of tetramer reagents).  Tetramers should be ~0.5 mg/mL solution.
Remove plate from incubator
Carefully draw off half of the media from each well
Transfer an aliquot of cells from each well - typically 75 μL or about 50,000 cells -  into a 5 mL polystyrene FACS tube
Add tetramer - typically 1 μL per 50 μL total volume - and place in 37°C incubator for 1-2  hours.  It is also advisable to stain a second aliquot of cells from each well with a mismatched tetramer as a negative control.
Label cells with anti-CD3, anti-CD4, and anti-CD25 antibodies by adding 3-10 μL of each antibody.  Additional or alternative antibody markers can be used.  However, do not use PE labeled antibodies since this channel must be reserved for the tetramers.  At this time, also label single color or isotype controls using extra cells.
Incubate antibody labeled cells for 15-30 minutes on ice or at 4°C.
Wash each tube with 0.5 to 2 mL running buffer and centrifuge at 230×g for 10 minutes.
Carefully decant liquid from FACS tubes.  Store all tubes in a covered container (preferably on ice) for subsequent FACS analysis.

### 5. Flow Cytometer Acquisition and Analysis

Calibrate the Flow Cytometer using reference beads.
Verify instrument settings:
  Using unstained control cells or isotype controls, adjust the location of the viable lymphocyte gate (FSC vs. SSC).  Remove auto-fluorescence from all channels by centering the population (by changing the gain settings) below the first decade on each axis.
  Using single color control tubes, center events within the correct quadrants for each single color (by changing the compensation settings).
  Do a fine adjustment of the instrument settings using a tube stained with an irrelevant tetramer.  In particular settings for the PE channel may need adjustment because tetramers often stain brighter than the antibody control.  The irrelevant tetramer should stain <0.5% of the CD4+ cells.
Acquire data for each tube
  Collect events for each sample tube and negative control tube
  Acquire and save at least 20,000 events for analysys
Analyze data
  Import data files into FACS analysis software (e.g CellQuest software, WinMDI, or FlowJo).  Start your analysis using a tube stained using an irrelevant tetramer.
  Plot FSC vs. SSC and draw a gate around the live lymphocytes (Figure 1, top left panel).
  Plot CD4 vs. CD3 (gated to show live lymphocytes only) and draw gates around the CD3+ and CD4+ populations (Figure 1 top right panel).
  Plot CD4 vs. Tetramer (gated to show CD3+ lymphocytes only) and set the quadrant boundaries so that the CD4+Tetramer+ population is less than 0.5% (Figure 1 bottom left panel).
  Plot CD25 vs. Tetramer (gated to show CD4+ lymphocytes only) and set the quadrant boundaries so that the CD25+Tetramer+ population is less than 0.5% (Figure 1 bottom right panel).
  Analyze all of the sample tubes without changing the gates.

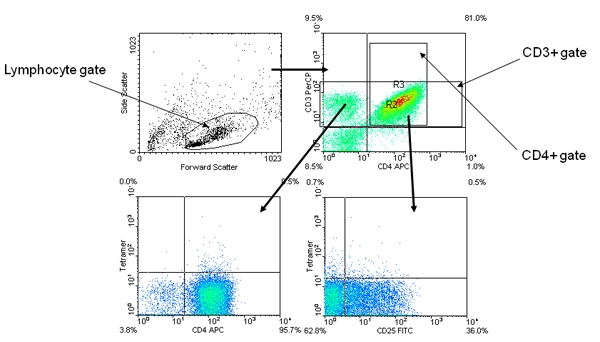


**Figure 1.** Representative negative control tetramer staining results.  The upper left panel shows forward scatter verses side scatter and the lymphocyte size gate (R1).  The upper right panel is gated for R1 and shows ant-CD4 versus anti-CD3 and the CD3+ (R2) and CD4+ (R3) gates.  The lower left panel is gated for R2 and shows anti-CD4 versus tetramer.  The lower right panel is gated for R3 and shows anti-CD25 versus tetramer.  The quadrants for both tetramer plots were set to 0.5%.

### 6. Representative Results

The tetramer positive cells should be a distinct population among the expanded CD4+ cells (Figure 2).
Tetramer positive cells are usually CD25+ and often CD4 high.
In some cases, a MHC/peptide combination can give background staining (often caused by a peptide with poor solubility).  Such background staining can be differentiated from a true positive because the “false positive” cells are not a single distinct population on either the CD4 vs. Tetramer or the CD25 vs. Tetramer plots (Figure 3).

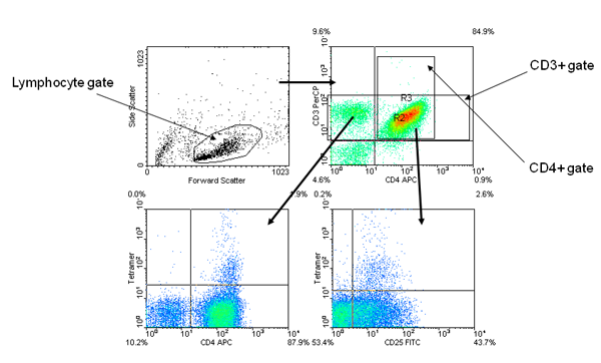


**Figure 2.** Representative positive tetramer staining results.  The panel layout and gating strategy are identical to Figure 1.  The tetramer positive cells appear as a distinct CD4 high population on the anti-CD4 versus tetramer panel and a distinct CD25+ population on the anti-CD25 versus tetramer panel.


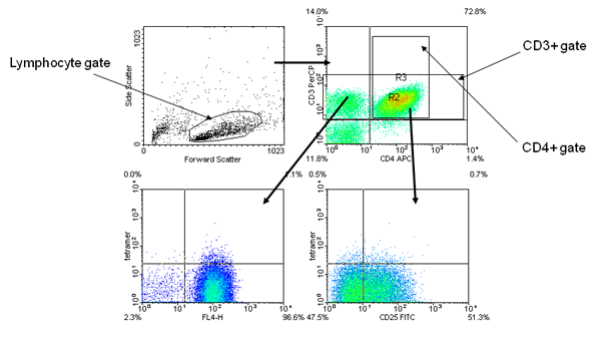


**Figure 3.** Representative “false positive” tetramer staining results.  The panel layout and gating strategy are identical to Figure 1.  The anti-CD4 versus tetramer panel shows an indistinct “mounded” staining. The anti-CD25 versus tetramer also shows a “mounded” staining, with no clear trend toward being CD25+.

## Discussion

Understanding the role of CD4+ T cells in immunity is fundamentally important.  However, antigen specific CD4+ T cells can be difficult to detect and isolate using traditional methods.  In contrast, MHC class II tetramers allow the direct visualization of CD4+ T cells with the desired antigen specificity.  This video demonstrates the isolation, purification, and in vitro amplification of CD4+ T cells and their subsequent visualization using tetramers.  Class II Tetramers are fluorescent protein conjugates consisting of soluble biotinylated class II α/β dimers conjugated around a fluorescent labeled streptavidin core (Figure 4). The tetramers used in this video were produced by the Teramer Core Laboratory at the Benaroya Research Institute using insect cell cultures (1).  These tetramers are prepared as a 0.5 mg/mL solution and can be stored at 4°C for 6-18 months.  Tetramers should never be frozen, as freeze-thaw stresses may strip the PE label away from the steptavidin core.  Class II staining reagents can be obtained from several sources (Table 1) and are designated as tetramers, “ultimers”, and multimers.  It should be noted that the tetramer assay procedure described here is distinct from the procedures used for class I tetramers.  Class I tetramer staining is usually optimal at 4°C, while class II tetramer staining generally requires 37°C.  Class I tetramers bind more tightly to T cells than Class II tetramers because of the stronger cooperative effect of CD8 as compared to CD4.  The tetramer assay described here is effective in visualizing T cell specific for either foreign or self antigens, but the strength of interaction is inherently lower for self antigens.  Several variations of the assay are possible.  For example, CD4+ T cells can be further fractionated prior to stimulation (e.g. into memory and naïve populations), stimulated using a whole protein or peptide pools (2), or various treatments may be added to different wells to measure their influence on the antigen specific response.  Since only a portion of each well is needed for tetramer analysis, the remaining cells can stained with tetramer for sorting or reserved for other purposes. The setup for each individual experiment will be dictated both by the characteristics of the sample and by the scientific question being asked.  For design purposes it is critical to know the HLA type of the blood donor because this will influence the epitopes used for expansion and will dictate the corresponding tetramer that must be used.  Knowledge of the disease or immunization status may also be crucial for interpreting results.  It is important to carefully maintain the cells during the amplification step, since unhealthy cells can give very poor results.  Also, proper machine setup, gating and negative controls are vitally important for obtaining high quality flow cytometry results.


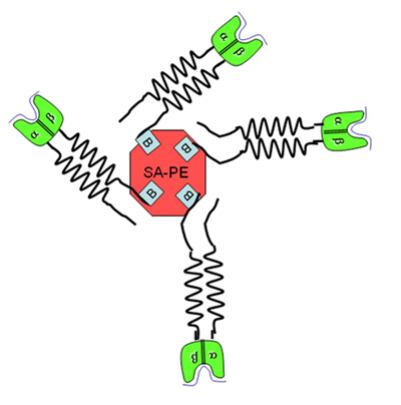


**Figure 4.** Schematic of MHC class II tetramer.  The red core represents the streptavidin-PE molecule.  The green and black extensions represent the MHC class II α/β dimers.  The complex is joined by the high affinity interaction between biotin and streptavidin.

### Table 1: Various sources of class II staining reagents

**Table:** 

**Tetramer Source**	**Product Type**	**Web Address**
Beckman Coulter	Tetramers	www.beckman.com
NIH Tetramer Facility	Tetramers	www.research.yerkes.emory.edu/tetramer_core
Proimmune	Ultimers	www.proimmune.com
Benaroya Research Institute	Multimers	www.benaroyaresearch.org
